# Catalogue Raisonné; or, Classified Arrangement of the Books in the Library of the Medical Society of Edinburgh

**Published:** 1837-04

**Authors:** 


Art. VI.—1. *Catalogue Raisonné; or, Classified Arrangement of the Books in the Library of the Medical Society of Edinburgh.—Edinburgh*, 1837. 8vo. pp. 342.

2. *An Address delivered to the Members of the Royal Medical Society (of Edinburgh,) December* 16*th*, 1836. By John H. Bennett, President of the Society. *Edinburgh*, 1837. 8vo. pp. 16.

We have no hesitation in saying that the catalogue now before us is the best medical catalogue in the English language, as the library of which it is the record, is one of the best in the kingdom. The general arrangement of the classes of works is excellent, while the minute subdivision of the subjects offers every possible facility to the student desirous of following up any branch of enquiry. In this respect the catalogue must prove of the greatest advantage, not merely to the young medical reader, but to every member of the profession who can have access to the library. In minuteness of subdivision of the subjects, it is more like Ersch's *Literatur der Medicin* than any other work we have met with; and it has the inestimable advantage over this or any other *general* catalogue, that every volume indicated is extant and accessible on the shelves of the library. We strongly recommend the councils of the College of Surgeons and of the Medico-Chirurgical Society of London, to arrange their libraries on the same plan: they cannot possibly follow a better model; nor can they confer a greater boon on the profession in London than by doing so. These libraries are much more extensive than the library of the Medical Society of Edinburgh, which however contains 12000 volumes. In this last we are struck with the very small number of German and Italian works, particularly the former: of French medical literature, on the contrary, there is a large stock.

It appears from the very excellent address of Mr. Bennett, that to the under-mentioned gentlemen* the society is indebted for this valuable work; and we are sure that the following account of their labours, extracted from the same discourse, is no exaggeration of their exertions or merits:—

"None but those who have engaged in such a task can in any way appreciate the enormous labour which the formation of our Catalogue Raisonné has occasioned. It must be remembered that this is not a work that could have been performed by a mere scrivener; it evinces literary, professional, and scientific knowledge in an eminent degree; while the judicious arrangement which characterizes it throughout, stamps on it the feature of originality. Figure to yourselves the labour and toil of reviewing separately the numerous works which compose our library,—of even writing their names. Add to this the trouble of classifying them, and the necessity of perusing many, the ambiguity of whose titles was such as not to denote their nature. Reflect on these circumstances, gentlemen, and consider that all this was undertaken without the hope of reaping any of those rewards for which most men enter into literary composition,—without the satisfaction even of having their names attached to their own publication: yet was this herculean labour cheerfully performed for the good and advantage of that institution endeared to them by the remembrance of the benefits it had conferred, and at whose shrine they thus offered up the most disinterested and undoubted proofs of devotion."

We have only one fault to find with this catalogue; and that is its title. The learned authors of it ought to have known that the epithet *Raisonné* is not strictly applicable to such a catalogue; and their taste ought to have prevented them from adopting a foreign word, when they could easily have found an English one to answer their purpose.

* Drs. Wood. Balfour, Maclagan, Dyer, A. Thomas, Imlach, G. Paterson, Ransford Taylor, and Mr. Seaton.

